# Microbial Co-occurrence Network and Fermentation Information of Natural Woody-Plant Silage Prepared With Grass and Crop By-Product in Southern Africa

**DOI:** 10.3389/fmicb.2022.756209

**Published:** 2022-03-14

**Authors:** Zhumei Du, Seishi Yamasaki, Tetsuji Oya, Damiao Nguluve, Denise Euridse, Benedito Tinga, Felicidade Macome, Yimin Cai

**Affiliations:** ^1^Japan International Research Center for Agricultural Sciences, Tsukuba, Japan; ^2^College of Grassland Science and Technology, China Agricultural University, Beijing, China; ^3^Agricultural Research Institute of Mozambique, Matola, Mozambique

**Keywords:** feed shortage, microbial co-occurrence network, natural biomass resource, silage fermentation, woody plant

## Abstract

To facilitate the use of woody plant (WP) as a natural biomass resource to address the shortage of feed for ruminants in the tropics, we use PacBio SMRT sequencing to explore the microbial co-occurrence network and silage fermentation of gliricidia and leucaena prepared with Napier grass (NG) and corn stover (CS) in Southern Africa. Based on dry matter, the crude protein contents of WP are as high as 25%. Compared with NG, the addition of CS speed up the dynamic succession of microorganisms in the silage fermentation process from Gram-negative bacteria to Gram-positive bacteria, and promoted *Lactiplantibacillus plantarum* to become the dominant community and enhanced the metabolic pathways of lactic acid and citric acid, thus improved the fermentation flavour and quality of WP silage. WP can be mixed with CS to make high-quality silage, which can alleviate the shortage of feed and promote local animal production.

## Introduction

To cope with insufficient feed caused by the rapid development of animal husbandry in the tropics, new and nutrient-rich locally available feed resources including natural woody plant (WP) have been investigated (Zhang et al., 2019). In addition to their contributions to a sustainable agricultural and animal ecosystem, tropical legume WP are rich sources of crude protein (CP) and minerals; they also promote biomass and animal production (Luscher et al., 2014).

Gliricidia [*Gliricidia sepium* (Jacq.) Kunth ex Walp.] is native to tropical, arid forests in Mexico and Central America. Beyond its region of origin, it is also grown in many tropical and subtropical regions, including the Caribbean, northern parts of South America, central Africa, portions of India, and Southeast Asia (Oliveira et al., 2018). Leucaena [*Leucaena leucocephala* (Lam.) de Wit] is a small, fast-growing mimosa tree native to southern Mexico and northern Central America (Belize and Guatemala), which has been naturalised throughout the tropics (Rengsirikul et al., 2011). The fresh branches and leaves of these WP are used as natural feed for grazing cattle because of their abundant nutrients, low lignin content, and good palatability (Speedy and Pugliese, 1992). Both types of WP have high biomass production capacity; the yield of their leaves can be as high as 20,000 kg-dry weight/ha/year. Under favourable climatic conditions, the yield can reach twice this level (Rajvanshi, 2019). Generally, the WP harvest time is concentrated in the rainy season, which is not conducive to hay preparation; however, silage fermentation is considered an ideal storage method. WP, like legume grass, have a high protein content, but they also have high moisture and low water-soluble carbohydrate (WSC) contents. Therefore, WP must be mixed with local gramineous forage or crop by-products to prepare silage.

Napier grass (NG, *Pennisetum purpureum* Schumach.) is the major feed resource in cattle production systems in Africa (Du et al., 2020). NG is widely planted in Africa because of its high biomass yield and adaptations for survival under a wide range of soil types, fertility levels, and weather conditions (Negawo et al., 2017). In contrast, corn (*Zea mays* L.) stover (CS) is the main crop by-product in Africa; dry stover is widely used for ruminant feed, but it is most often discarded in the field to be burned and used as fertiliser (Cai et al., 2020a). NG and CS are used to adjust the moisture content and increase the fermentation substrate level to optimise WP silage fermentation.

PacBio Single-Molecule Real-Time (SMRT) sequencing technology can cover the full read length of DNA fragments and obtain long sequence reads, providing microbial diversity information at the species level. This technology has been used to study the microbial community and fermentation mechanism in silage. However, there is limited information on how to improve the fermentation quality of WP silage in combination with local forage resources in Africa. To develop a high-quality WP preparation technology to alleviate the shortage of feed for ruminants and evaluate silage-related microbial communities, we used SMRT sequencing to assess the microbial community and co-occurrence network related to silage fermentation of WP prepared with NG and CS in Southern Africa.

## Materials and Methods

### Woody Plant and Silage Fermentation

The experiment was conducted at an experimental farm of the Agricultural Research Institute of Mozambique (IIAM, Matola, Mozambique) on February 26, 2019. NG was harvested from the first cutting at the early flowering stage. Corn was cultivated by a local farm in the same area, and fresh CS was collected after the harvest of corn cobs were harvested during the experiment. Gliricidia and leucaena grow naturally in hilly areas in the region (Matola, Mozambique). Young branches and leaves of both WP at the juvenile stage were obtained from first cuttings in different hilly areas with three replicates.

**FIGURE 1:**
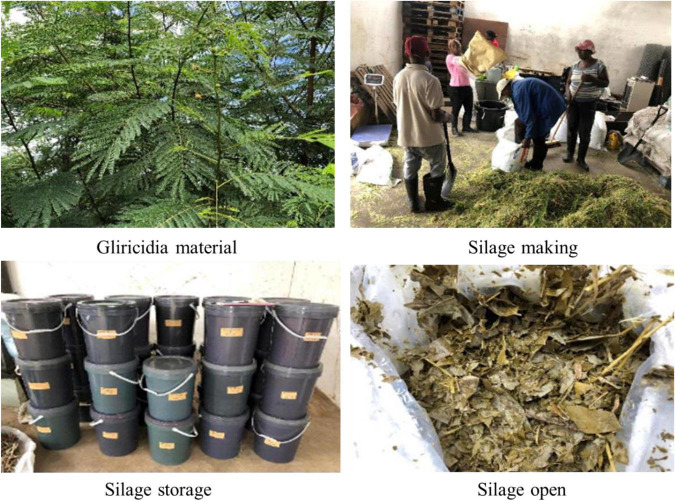
Silage preparation of woody plant.

After harvest, gliricidia, leucaena, NG, and CS were immediately chopped into lengths of approximately 1–2 cm using a chopper (130DX; ARS Co., Ltd., Osaka, Japan). The chopped materials were homogenised according to the experimental design and the silages were prepared with gliricidia, leucaena, NG, CS, and their mixture. The mixing proportions of WP with NG or CS are 10, 25, and 50% based on a fresh matter (FM) basis. These homogenised materials were divided into two fractions. The first fraction was collected as fresh samples and placed into sterilised bags that were kept in an ice box. These were immediately transported to a laboratory to determine their lactic acid buffering capacity (LBC), chemical and protein compositions, energy, macro-minerals, and microbial community. The second fraction was used for making silage. As shown in Figure 1, the silages were prepared with three replicates by using polyethylene drum silos (20 L, Ka-Kosher Co., Ltd., Sinaloa, Mexico). Approximately 16 kg of mixture material was packed into the silo, all of the silos were then compacted to exclude air and storage was at ambient temperature (24–37°C). After 60 days of ensiling, silage samples (approximately 500 g) from each replicate from the top, middle, and bottom layers of the drum silos were taken and mixed thoroughly before taking subsamples. The fresh samples in each treatment were divided into three portions. The first is to store the samples (approximately 50 g) in a freezer at −80°C for future analysis of microbial community; the second is to dry the samples (approximately 200 g) for subsequent analysis of dry matter (DM), chemical and protein compositions, CP loss; and the third is to use 10 g samples to prepare the extract liquid to analyse the microbial population and fermentation quality of the silage.

### Microbial Analysis

The microbial population of materials and silages consisted of lactic acid bacteria (LAB), aerobic bacteria, coliform bacteria, yeasts, and moulds, which were measured by the plate counting method described by Cai et al. (1999). Samples (10 g) were blended with 90 mL of sterilised saline solution (8.50 g/L NaCl) and homogenised for 5 min in a Stomacher lab blender (400; Seward, United Kingdom). The resulting suspension was serially diluted from 10^–1^ to 10^–8^ with saline solution. A 0.05 mL aliquot from each diluted suspension was spread on agar plates. LAB were counted on Lactobacilli de Man, Rogosa, and Sharpe (MRS) agar medium (Difco Laboratories, Detroit, MI, United States) in an anaerobic box (TEHER Hard Anaerobox, ANX-1; Hirosawa Ltd., Tokyo, Japan). The LAB were identified by the Gram-positive and catalase-negative rods or cocci that produced lactic acid. Aerobic bacteria were grown on nutrient agar medium (Nissui-Seiyaku Co., Ltd., Tokyo, Japan) under aerobic conditions. Coliform bacteria were counted on blue light broth agar medium (Nissui-Seiyaku Co., Ltd., Tokyo, Japan), and their colonies were distinguished from other bacteria by the blue-colour colonies. All bacterial agar plates were incubated at 30°C for 2–3 days. Yeasts and moulds were counted on potato dextrose agar medium (Nissui-Seiyaku Co., Ltd., Tokyo, Japan) with sterilised tartaric acid solution (pH 3.5) at 30°C for 3–5 days of incubation. Yeasts and moulds were distinguished from other bacteria based on colony appearance and cell morphology. All the microbial colonies were reported as viable microbial numbers in colony-forming unit (cfu)/g of FM.

For SMRT sequencing analysis, the triplicate samples (10 g) were mixed with 90 mL of sterilised 0.85% NaCl solution and shaken at the speed of 250 rpm in a 4°C refrigerator for 45 min. The liquid mixture was filtered through a four-layer cheesecloth pre-autoclaved, and then the filtrate was centrifuged at 10,000 rmp for 10 min at 4°C to obtain microbial precipitate. Then the precipitated sample was used for DNA extraction by a DNA kit (D5625-01, Omega, Norcross, GA, United States) as described by Du et al. (2021a). The quality of the extracted DNA was monitored on 1% agarose gel electrophoresis and spectrophotometry (optical density at 260/280 nm ratio). All the DNA samples were stored at −20°C for future analysis.

The forward primer 27F and reverse primer 1492R were used to amplify the full-length 16S rRNA gene by PCR for SMRT sequencing (Du et al., 2021b). The PCR amplicons were purified using Agencourt AMPure XP Beads (Beckman Coulter, Indianapolis, IN, United States), and quantified by Qubit dsDNA HS Assay Kit and Qubit 3.0 fluorometer (Invitrogen, Thermo Fisher Scientific, Waltham, MA, United States). After the individual quantification step, amplicons were pooled in equal amounts. SMRTbell libraries were prepared from the amplified DNA by SMRTbell Express Template Prep Kit 2.0 according to the manufacturer’s instructions (PacBio, Menlo Park, CA, United States). Purified SMRTbell libraries from the pooled and barcoded samples were sequenced on a single PacBio Sequel II 8M cell using the Sequel II Sequencing kit 2.0.

Single-Molecule Real-Time sequencing was performed on a PacBio RS II instrument (Pacific Biosciences, Menlo Park, CA, United States) using P6-C4 chemistry (Mosher et al., 2013). Raw data were processed using the protocol RS_Readsofinsert.1 in SMRT Portal version 2.7 software (PacBio) (Du et al., 2021a). Low-quality sequences were removed using the Quantitative Insights Into Microbial Ecology (QIIME) package (version 1.7) (Caporaso et al., 2010a). Using 100% clustering of sequence identity, the extracted high-quality circular consensus sequence (CCS) were aligned to obtain representative sequences using Python nearest alignment space termination (PyNAST) and clustering and classification inference with U-statistics (UCLUST) analysis (Caporaso et al., 2010b; Edgar, 2010). Unique sequences were classified into operational taxonomic unit (OTU) based on a 99% threshold identity using the UCLUST algorithm (Lozupone and Knight, 2005). Potential chimeric sequences in the representative set of OTU were removed using the Chimera Slayer tool (Haas et al., 2011). The SILVA database version 132 was implemented to classify different OTU and annotate the taxonomic information for each OTU representative sequence based on Bergey’s taxonomy at the genus, family, order, class, and phylum levels, according to classification at an 80% minimum bootstrap threshold (Quast et al., 2013). OTU that occurred only once or twice were discarded. In order to describe the shared and unique microorganisms in all samples following the OTU clustering analyses, Venn diagrams were produced using open-source software package (version 1.2) of R statistical tools (Shade and Handselman, 2012). Since mixture silages showed similar bacterial community, we used gliricidia or leucaena + NG or CS (90 + 10) mixed silages as a representative for Venn diagram analysis. The relative abundances of different bacterial communities at the species level were also analysed for Windows statistical software package. Hierarchical cluster and heat map analyses were performed using R package pvclust (version 3.0.2) (Suzuki and Shimodaira, 2006; Mansfeldt et al., 2014). A microbial network analysis was drawn with Python language tool (Langfelder and Horvath, 2008).

The metabolic potential of the microbial community and the composition of functional genes were assigned to functional annotations of sequenced metagenomic sequences through 16S rRNA marker gene, and were postulated based on the Kyoto Encyclopedia of Genes and Genomes (KEGG). KEGG is utilised for bioinformatics research and education, including data analysis in genomics, metagenomics, metabolomics and other omics studies, modelling and simulation in systems biology, and translational research in drug development. The functional profiles and differences among different groups were analysed with phylogenetic investigation of communities by reconstruction of unobserved states (PICRUSt2) (Langille et al., 2013). Due to the same reason as the Venn diagram analysis, that is, two types of woody silage have similar fermentation characteristics, we only use gliricidia material and silage, and gliricidia silage and gliricidia + CS (90 + 10) mixed silage for metabolic pathway analysis.

### Chemical Analysis

The pre-ensiled materials and the silage were dried in an oven for 48 h at 65°C until a constant mass was attained. After drying, the samples were ground using a high speed vibrating sample mill (T1-200; for use with two containers with a working capacity of 50 mL; CMT Japan Co., Ltd., Yokohama, Japan). According to the methods of the AOAC International (2000), samples were analysed for DM (method 930.15), ash (method 923.03), CP (method 990.03), and ether extract (EE, method 920.39). The organic matter (OM) content was calculated as the weight lost after ashing. Neutral detergent fibre (NDF), acid detergent fibre (ADF), and acid detergent lignin (ADL) were determined as described by Van Soest et al. (1991). The NDF and ADF were expressed exclusive of residual ash. Heat stable amylase and sodium sulphite were used for the NDF procedure. ADL was analysed by solubilisation of cellulose with sulphuric acid. LBC was determined by titrating with NaOH from pH 4.0 to 6.0 (mmol/kg DM) after first reducing the pH to below 4.0 using HCl, as described by Muck et al. (1991). The WSC including glucose, sucrose, and fructose were determined by high performance liquid chromatography (HPLC, LC-2000 plus; JASCO Co., Tokyo, Japan) as described by Cai (2004). The analytical conditions were as follows: column, SC 1011 (8.0 mm × 30 cm, Shoko, Tokyo, Japan); oven temperature, 80°C; mobile phase, water; flow velocity, 1.0 mL/min; and detector, Jasco RI-1530.

Binding protein, effective protein, and neutral detergent insoluble protein (NDIP, indicates the CP contents of NDF residue) were analysed by the method of Cai et al. (2020b). CP loss (%) = (CP content of fresh material–CP content of silage)/CP content of fresh material × 100%. Gross energy (GE) was determined using an automatic oxygen bomb calorimeter (CA-4PJ; Shimadzu, Kyoto, Japan) (Gao et al., 2019). Digestible energy (DE) and metabolisable energy (ME) concentrations of the test ingredients were calculated using a difference procedure (Baker and Stein, 2009; Li et al., 2018). Herein, CP, EE, NDF, ADF, and ash are expressed as a percentage of DM, and the units for GE, DE, and ME are MJ/kg of DM. The macro-minerals contents of samples, including calcium, phosphorous, magnesium, and potassium, were measured using a wet-digestion method, and then analysed with an atomic absorption spectrophotometer (PerkinElmer, LAMBDA 1050, Yokohama, Japan) as described by Pequerul et al. (1993).

### Analysis of Silage Fermentation

The terminal fermentation products of the silages were analysed using the method of cold-water extracts, as described by Cai (2004). The remaining wet silage sample (10 g) was homogenised in 90 mL of sterilised distilled water and kept in a refrigerator at 4°C for 24 h. Thereafter, the extract samples were filtered through quantitative ashless filter paper (circle size: 5A, 110 mm; Advantec Co., Ltd., Tokyo, Japan). The filtrate was used to determine pH, ammonia nitrogen (NH_3_-N), and organic acid (lactic acid, acetic acid, propionic acid, and butyric acid) contents. The pH was measured using a glass electrode pH meter (D-71; Horiba Co., Ltd., Kyoto, Japan). The NH_3_-N contents of silages were determined by steam distillation of the filtrates, as described by Cai (2004) using Kjeltec auto distillation (2200; Foss Tecator, Höganäs, Sweden). The silage filtrates were shaken with cation exchange resin (Amberlite, IR 120B H AG; Organo Corporation, Tokyo, Japan) and centrifuged at 6,500 × *g* and 4°C for 5 min. The supernatants were passed through a 0.45 mm filter under pressure, and the filtrates were then injected into an HPLC system (LC-2000 plus; JASCO Co., Tokyo, Japan) to determine organic acid contents in accordance with the methods described by Cai (2004). The HPLC system was equipped with a Shodex RSpak KC-811 column (8.0 mm × 30 cm; Showa Denko K. K., Tokyo, Japan) at an oven temperature of 60°C. The detector was a Jasco UV-2070 used at 450 nm with an eluent of 3 m*M* HClO_4_ and reagent of 0.2 m*M* bromothymol blue + 8 m*M* Na_2_HPO_4_ + 2 m*M* NaOH. The flow rate was 1.0 mL/min.

### Statistical Analysis

ANOVA was performed using the general linear model (GLM) procedure of Statistical Package for the Social Sciences (SPSS Version 19.0, SPSS Inc., Chicago, IL, United States) to examine the differences between samples, and significance was declared at *P* < 0.05. The LBC, microbial population, chemical and protein compositions, energy, and macro-minerals contents of samples were subjected to one-way ANOVA. Tukey’s honest significant difference (HSD) test was employed for different sample means (Steel and Torrie, 1980).

The hierarchical cluster and heat map analyses showed the correlation analyses of the bacterial community with lactic acid, LAB, pH, and NH_3_-N at species level, respectively. LAB, organic acid, pH, and NH_3_-N information are displayed horizontally, respectively, and the bacterial community information is displayed vertically. The corresponding value of the middle heat map is the Spearman correlation coefficient r, which ranges between −1 and 1, *r* < 0 indicates a negative correlation (blue), *r* > 0 indicates a positive correlation (red), and “*,” “^**^,” and “^***^” represent *P* < 0.05, *P* < 0.01, and *P* < 0.001, respectively. The network analysis showed the correlation networks among microorganisms at species levels. The circle represents the microorganism species, the circle size represents the average abundance of the species, the line represents the correlation between the two species, the thickness of the line represents the strength of the correlation, and the colour of the line: orange represents positive correlation, green means negative correlation.

## Results

The pH, LBC, microbial population, chemical and protein compositions, energy, and macro-mineral values of WP, NG, and CS before ensiling are shown in Table 1. The WSC content was lower (*P* < 0.001) and the LBC was higher (*P* < 0.001) in WP than in both forages. The WSC content was significantly lower (*P* < 0.001) in NG than in CS, but LBC showed the opposite pattern (*P* < 0.01). The LAB count was higher (*P* < 0.001) in CS than in WP and NG. Aerobic bacteria were predominant in all samples, and their counts were higher (all *P* < 0.05) than the counts of other microorganisms. The yeast count was lower (*P* < 0.05) in WP than in forages, whereas coliform bacteria showed the opposite pattern. The mould counts of all samples were similar at 3 lg cfu/g of FM. The DM contents in WP, NG, and CS ranged from 21.18 to 38.94%. The CP contents in gliricidia and leucaena were >25.91% of the contents in DM; the CP contents were higher (*P* < 0.001) in gliricidia and leucaena than in NG and CS. The EE and ADL contents were higher (*P* < 0.001) in WP than in NG and CS, but the NDF and ADF contents showed the opposite pattern. The effective protein, GE, DE, ME, calcium, magnesium, and potassium contents were higher (all *P* < 0.001) in gliricidia and leucaena than in NG and CS, whereas the binding protein and NDIP contents showed the opposite pattern. The phosphorous contents were similar in all samples.

**TABLE 1:** pH, LBC, microbial population, chemical and protein compositions, energy, and macro-mineral of material.

Items	Gliricidia	Leucaena	NG	CS	*P*-value
pH	6.38*b*	6.31*b*	6.47*a*	6.60*a*	0.02
LBC (mEq/kg of DM)	577.16*a*	508.18*a*	332.10*b*	266.62*c*	<0.001
**Microbial population (lg cfu/g of FM)**					
Lactic acid bacteria	4.04*b*	4.02*b*	4.00*b*	5.14*a*	<0.001
Aerobic bacteria	8.28*a*	8.10*a*	7.33*b*	7.43*b*	0.03
Coliform bacteria	5.73*b*	5.66*b*	3.88*c*	6.11*a*	0.04
Yeast	4.51*b*	4.44*b*	5.35*a*	5.45*a*	0.04
Mould	3.07	3.13	3.22	3.37	0.06
**Chemical composition**					
DM (%)	24.92*b*	21.18*b*	38.94*a*	36.88*a*	<0.001
OM (% of DM)	90.30*d*	92.62*b*	91.19*c*	93.26*a*	<0.001
CP (% of DM)	25.91*b*	26.31*a*	7.99*c*	7.27*c*	<0.001
EE (% of DM)	4.02*a*	3.41*b*	2.69*c*	1.85*d*	<0.001
NDF (% of DM)	52.10*c*	60.62*b*	66.74*a*	65.05*a*	<0.001
ADF (% of DM)	34.52*d*	37.49*c*	45.92*a*	40.53*b*	<0.001
ADL (% of DM)	11.09*b*	13.40*a*	5.03*c*	4.53*d*	<0.001
WSC (% of DM)	4.62*c*	4.97*c*	5.53*b*	10.48*a*	<0.001
**Protein composition (% of CP)**					
Binding protein	13.64*c*	16.60*b*	23.98*a*	22.64*a*	<0.001
NDIP	37.40*b*	34.69*c*	42.20*a*	42.76*a*	<0.001
Effective protein	76.77*a*	74.95*b*	69.01*c*	68.81*c*	<0.001
**Energy (MJ/kg of DM)**					
GE	20.07*a*	20.94*a*	14.03*c*	15.44*b*	<0.001
DE	12.55*a*	11.62*b*	10.01*c*	10.73*c*	<0.001
ME	9.47*a*	9.53*a*	8.07*b*	8.30*b*	<0.001
**Macro-mineral (g/kg of DM)**					
Calcium	1.57*a*	1.26*b*	0.84*c*	0.53*c*	<0.001
Phosphorous	0.27	0.23	0.42	0.19	0.06
Magnesium	0.59*a*	0.42*b*	0.32*c*	0.20*c*	<0.001
Potassium	2.64*a*	2.56*b*	2.42*c*	1.69*d*	<0.001

*a–d: Data are means of three samples, means in the same row followed by different letters differ (P < 0.05).*

*NG, Napier grass; CS, corn stover; LBC, lactic acid buffering capacity; DM, dry matter; cfu, colony-forming unit; FM, fresh matter; OM, organic matter; CP, crude protein; EE, ether extract; NDF, neutral detergent fibre; ADF, acid detergent fibre; ADL, acid detergent lignin; WSC, water-soluble carbohydrate; NDIP, neutral detergent insoluble protein; GE, gross energy; DE, digestible energy; ME, metabolisable energy.*

The chemical and protein compositions and CP loss values of WP, NG, CS, and their mixture silages are shown in Table 2. For the WP mixture silages, as the proportion of NG or CS increased, the OM, CP, EE, and ADL contents decreased; conversely, the DM, NDF, and ADF contents and CP loss increased. The WP, NG, and their mixture silages had higher (all *P* < 0.05) CP losses than did the CS and its mixture silages.

**TABLE 2:** Chemical and protein compositions, and CP loss of WP, NG, CS, and their mixture silages.

Items	DM (%)	Chemical composition (% of DM)						CP loss (%)
		OM	CP	EE	NDF	ADF	ADL	
Gliricidia	21.34d	90.89e	23.20a	3.67a	50.02e	33.91g	10.24b	19.70a
Leucaena	20.31d	91.36c	24.82a	3.32b	57.36b	34.99f	13.39a	19.32a
NG	38.78c	90.11f	6.52f	1.77h	64.01a	44.70a	4.72i	18.40a
CS	37.25c	91.40c	6.47f	1.78h	63.58a	38.89e	3.87j	5.36b
**Gliricidia + NG**								
90 + 10	32.54c	91.10d	18.65c	3.14c	53.08c	40.22c	8.06e	19.04a
75 + 25	47.94b	90.97e	15.26d	2.46f	55.28c	43.07b	6.55g	19.42a
50 + 50	56.93a	90.75e	13.48e	2.09g	59.42b	44.78a	5.64h	19.65a
**Gliricidia + CS**								
90 + 10	31.65c	92.98a	20.89b	3.36b	51.81d	37.93ef	8.27de	6.09b
75 + 25	43.79b	92.56a	18.59c	3.02d	53.76c	39.23d	6.40g	6.28b
50 + 50	52.71a	91.87b	15.14d	2.82de	58.58b	40.04c	5.28g	6.60b
**Leucaena + NG**								
90 + 10	34.81c	91.44c	17.27c	2.69e	53.68c	41.48c	9.35c	18.82a
75 + 25	45.21b	91.64b	14.54d	2.23f	55.21c	43.47b	7.32f	19.40a
50 + 50	55.87a	91.97b	12.86e	1.95g	56.13b	44.66a	6.10gh	19.61a
**Leucaena + CS**								
90 + 10	36.67c	92.91a	21.53b	3.19c	52.84d	38.97e	8.95d	6.12b
75 + 25	45.66b	92.87a	19.03c	3.00d	53.94c	39.95cd	6.58g	6.76b
50 + 50	53.97a	92.85a	14.86d	2.92de	54.61bc	40.53c	5.16h	6.84b
SEM	0.14	0.20	1.46	0.17	0.86	0.91	0.22	0.39
*P*-value	<0.001	<0.001	<0.001	<0.001	<0.001	<0.001	<0.001	<0.001

*a–j: Data are means of three samples, means in the same column followed by different letters differ (P < 0.05).*

*90 + 10, 75 + 25, 50 + 50, indicated the mixing ratio (%) of silage based on fresh matter; CP, crude protein; WP, woody plant; NG, Napier grass; CS, corn stover; DM, dry matter; OM, organic matter; EE, ether extract; NDF, neutral detergent fibre; ADF, acid detergent fibre; ADL, acid detergent lignin; NDIP, neutral detergent insoluble protein; SEM, standard error of the mean.*

The microbial population and fermentation quality of WP, NG, CS, and their mixture silages are shown in Table 3. Moulds were below the detectable level (<2 lg cfu/g of FM). LAB (7 lg cfu/g of FM) was predominant in CS and its mixture silages prepared with WP; LAB values were higher (all *P* < 0.05) in these samples than in other silages. Aerobic bacteria and coliform bacteria were present in WP, NG, and their mixture silages at 4–7 lg cfu/g of FM, but they were below detectable levels in CS and its mixture silages. Compared with NG, CS and its mixture silages had better fermentation quality with a lower (*P* < 0.001) pH and lower (both *P* < 0.001) acetic acid and NH_3_-N contents, as well as a higher (*P* < 0.05) lactic acid content. Propionic acid and butyric acid were produced in WP, NG, and their mixture silages, but they were below detectable levels in CS and its mixture silages.

**TABLE 3:** Microbial population and fermentation quality of WP, NG, CS, and their mixture silages.

Items	Microbial population (lg cfu/g of FM)					pH	Organic acid (% of FM)				NH_3_-N (% of DM)
	LAB	Aerobic bacteria	Coliform bacteria	Yeast	Mould		Lactic acid	Acetic acid	Propionic acid	Butyric acid	
Gliricidia	6.52e	5.39c	5.87b	ND	ND	5.40b	0.28d	0.30c	0.04b	0.28c	0.42c
Leucaena	6.54e	6.91ab	4.98c	5.32a	ND	5.83a	0.30d	0.24d	0.05b	0.35b	0.35d
NG	6.54e	7.35a	6.06a	5.49a	ND	4.87e	0.45c	0.53a	0.12a	0.46a	0.58a
CS	7.58b	ND	ND	5.31a	ND	4.09f	1.44a	0.34c	ND	ND	0.26e
**Gliricidia + NG**											
90 + 10	6.83d	7.33a	5.44b	5.21a	ND	5.04d	0.41c	0.35c	0.03b	0.43a	0.55a
75 + 25	6.68d	6.64b	6.21a	4.31b	ND	5.22d	0.30d	0.45b	0.06b	0.35b	0.50b
50 + 50	6.60e	6.36b	6.75a	4.22b	ND	5.35c	0.35d	0.50a	0.10a	0.30b	0.44c
**Gliricidia + CS**											
90 + 10	7.90a	ND	ND	5.75a	ND	3.72h	1.45a	0.34c	ND	ND	0.41c
75 + 25	7.68b	ND	ND	4.79b	ND	3.97g	1.10b	0.37b	ND	ND	0.35d
50 + 50	7.59b	ND	ND	ND	ND	4.01f	0.72c	0.39b	ND	ND	0.29e
**Leucaena + NG**											
90 + 10	6.97d	6.33b	4.84c	ND	ND	5.48b	0.44c	0.29d	0.05b	0.46a	0.51b
75 + 25	6.58e	5.00d	5.41b	ND	ND	5.69b	0.37d	0.42b	0.07a	0.41a	0.45c
50 + 50	6.05f	5.00d	5.49b	ND	ND	5.83a	0.31d	0.50a	0.11a	0.38b	0.37d
**Leucaena + CS**											
90 + 10	7.82a	ND	ND	4.80b	ND	4.01f	1.45a	0.25d	ND	ND	0.27e
75 + 25	7.57b	ND	ND	ND	ND	4.38ef	0.83b	0.27d	ND	ND	0.32d
50 + 50	7.16c	ND	ND	ND	ND	4.47ef	0.54c	0.32c	ND	ND	0.46c
SEM	0.30	0.36	0.54	0.29	NS	0.15	0.06	0.05	0.02	0.03	0.02
*P*-value	<0.001	<0.001	<0.001	<0.001	NS	<0.001	<0.001	0.02	<0.001	<0.001	<0.001

*a–h: Data are means of three samples, means in the same column followed by different letters differ (P < 0.05).*

*90 + 10, 75 + 25, 50 + 50, indicated the mixing ratio (%) of silage based on fresh matter; WP, woody plant; cfu, colony-forming unit; FM, fresh matter; LAB, lactic acid bacteria; NH_3_-N, ammonia nitrogen; NG, Napier grass; CS, corn stover; DM, dry matter; SEM, standard error of the mean; ND, not detected.*

**FIGURE 2:**
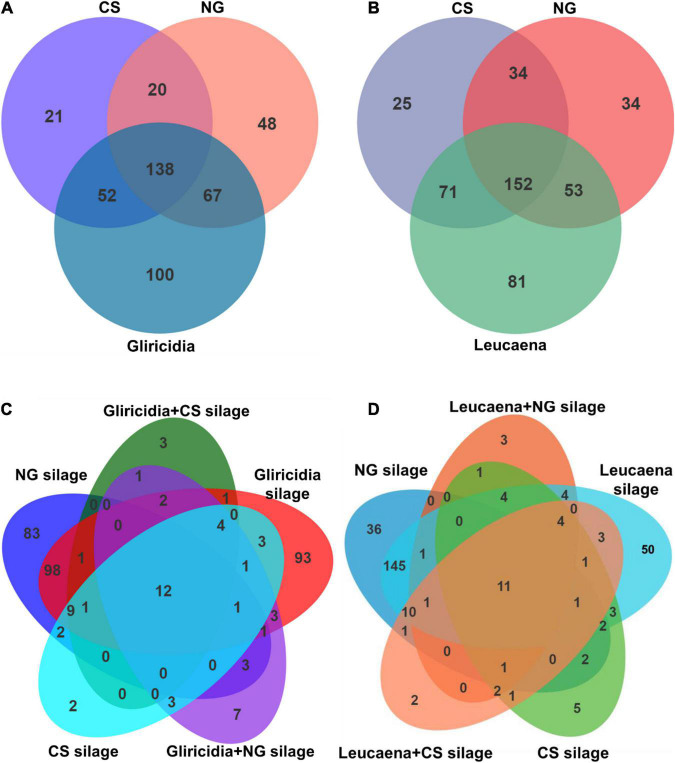
Venn diagram depicting unique or shared bacterial OTU (97% sequence identity) in gliricidia, leucaena, NG, CS materials and their mixture silages. **(A)** The number of OTU shared by gliricidia, NG, and CS before ensiling. **(B)** The number of OTU shared by leucaena, NG, and CS before ensiling. **(C)** The number of OTU shared by gliricidia, NG, and CS and their mixture silages. **(D)** The number of OTU shared by leucaena, NG, and CS and their mixture silages. OTU, operational taxonomic unit; NG, Napier grass; CS, corn stover. The mixing ratio of gliricidia or leucaena were 90% of the mixture silage based on a fresh matter.

A Venn diagram of the OTU at 97% sequence identity in WP prepared with NG and CS before and after ensiling is shown in Figure 2. The dominant microbiome of the gliricidia, NG, and CS materials (Figure 2A) contained 138 shared OTU, as well as 21, 48, and 100 unique OTU, respectively. The leucaena, NG, and CS materials (Figure 2B) contained 152 shared OTU and 25, 34, and 81 unique OTU, respectively. The WP silages shared 12 and 11 OTU with the gliricidia (Figure 2C) and Leucaena (Figure 2D) silages prepared with NG and CS, respectively. As seen in Figure 2C, the unique OTU ranged from 2 to 93 in CS, NG, gliricidia, and their mixture silages. As seen in Figure 2D, the unique OTU ranged from 2 to 50 in CS, NG, leucaena, and their mixture silages.

**FIGURE 3:**
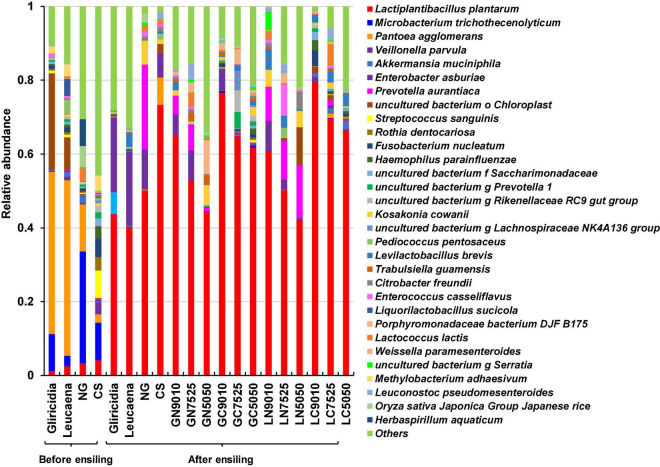
The relative abundances of the bacterial communities at the species levels in the gliricidia, Leucaena, NG, and CS materials, and their mixture silages. NG, Napier grass; CS, corn stover; GN, gliricidia + NG; GC, gliricidia + CS; LN, leucaena + NG; LC, leucaena + CS; 9010, 90 + 10%; 8020, 80 + 20%; 7030, 70 + 30%.

Relative bacterial abundances at the species level in the WP, NG, and CS materials, and their mixture silages are shown in Figure 3. The dominant species in the WP material was *Pantoea agglomerans*. The dominant species in the NG material were *Microbacterium trichothecenolyticum* and *P. agglomerans*; the dominant species in CS material were *M. trichothecenolyticum*, *Streptococcus sanguinis*, and *Methylobacterium adhaesivum*. *Lactiplantibacillus plantarum* was present at a low level in all materials. After ensiling, *L. plantarum* was the predominant species in all silages, and its counts were higher (both *P* < 0.05) in NG and CS silages than in WP silage; CS silage had the highest number. Compared with the WP silages, the addition of NG and CS significantly increased (both *P* < 0.05) the proportion of *L. plantarum* in the mixture silages, with the highest relative abundances in the WP and CS mixture silages.

**FIGURE 4:**
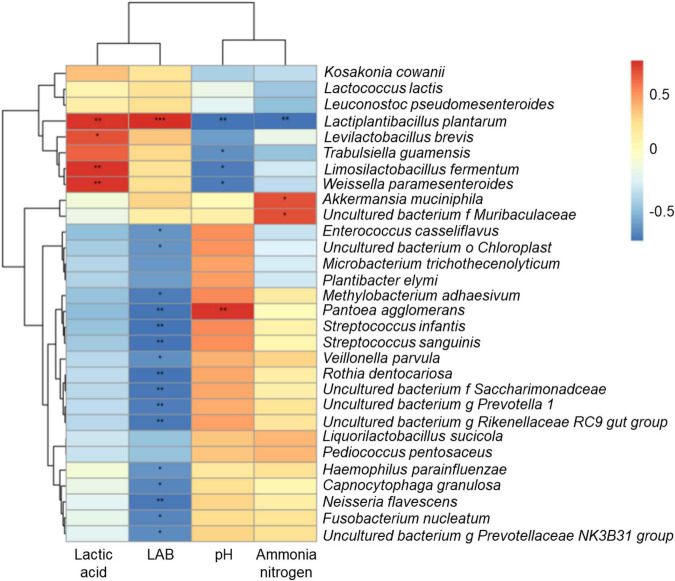
Correlation heatmap and hierarchical cluster analysis between bacterial community and terminal fermentation products at species level. LAB, lactic acid bacteria; NH_3_-N, ammonia nitrogen. *P < 0.05; **P < 0.01; ***P < 0.001.

Correlation heatmap and hierarchical cluster analysis of the bacterial community at the species level and terminal fermentation products are shown in Figure 4. Lactic acid was positively correlated with *Lactobacillus* and *Weissella* species, such as *L. plantarum*, *Levilactobacillus brevis*, *Limosilactobacillus fermentum*, and *Weissella paramesenteroides*. LAB was positively correlated with *L. plantarum*, but negatively correlated with *P. agglomerans*. pH showed the opposite patterns. NH_3_-N content was negatively correlated with *L. plantarum* and positively correlated with *Akkermansia muciniphila*.

**FIGURE 5:**
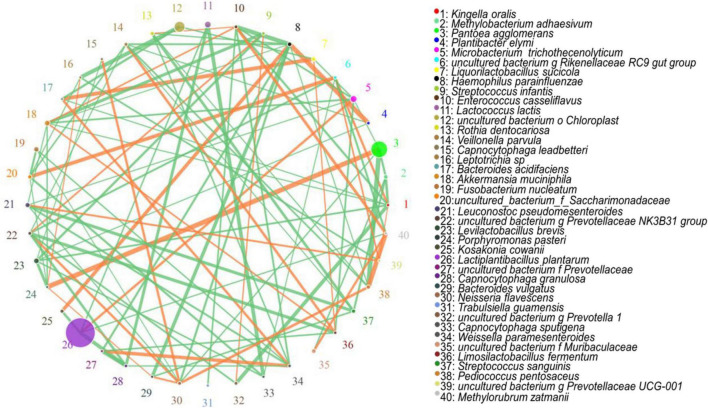
Correlation networks among all the microorganisms at the species level.

The correlation networks among all microorganisms at the species level are shown in Figure 5. The most important species appeared to be *L. plantarum*, which was positively correlated with *M. trichothecenolyticum* and negatively correlated with both *Kosakonia cowanii* and *Capnocytophaga granulosa*. The second most important species was *P. agglomerans*, which was positively correlated with *Porphyromonas pasteri* and negatively correlated with *M. adhaesivum*.

**FIGURE 6:**
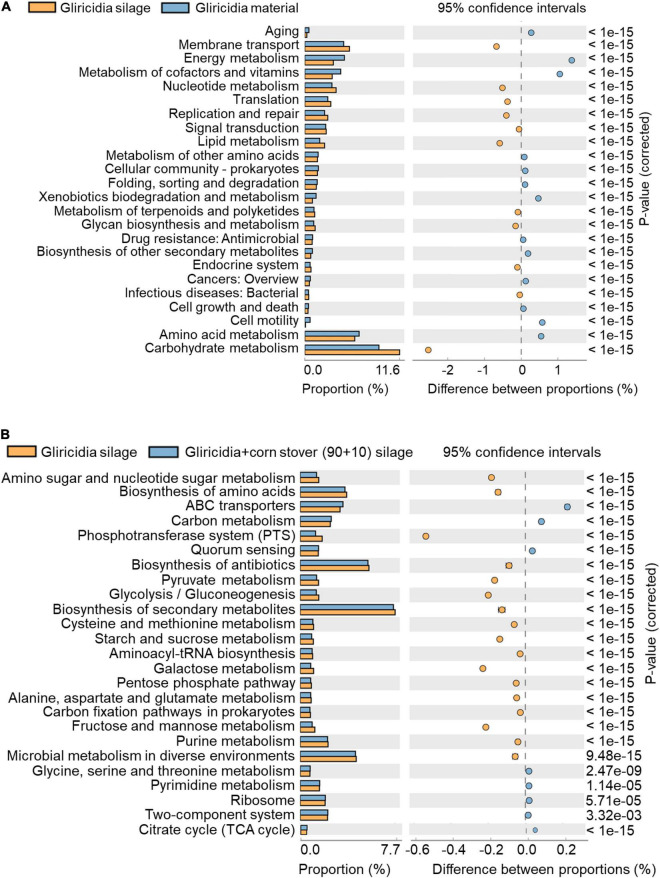
KEGG metabolic pathways in gliricidia material and silage **(A)**, and gliricidia silage and gliricidia + CS (90 + 10) mixed silage **(B)**. KEGG, Kyoto Encyclopedia of Genes and Genomes.

The KEGG pathways enriched in gliricidia material and silage are shown in Figure 6A; the KEGG pathways enriched in gliricidia silage and gliricidia + CS mixed silage are shown in Figure 6B. Figure 6A shows that the carbohydrate metabolism, amino acid metabolism, and energy metabolism pathways were the predominant metabolic categories. Among them, the largest proportion of the metabolism pathway involved carbohydrate metabolism. The proportion of the carbohydrate metabolism pathway was higher (*P* < 0.01), whereas the proportions of amino acid and energy metabolism pathways were lower (both *P* < 0.01) in gliricidia silage than in gliricidia material. Figure 6B shows that the biosynthesis of secondary metabolites, biosynthesis of antibiotics, and microbial metabolism in diverse environments were the predominant metabolic categories in gliricidia silage and mixture silages. Compared with the gliricidia silage, the mixture silage had lower (*P* < 0.01) proportions of the above three metabolic pathways and a higher (*P* < 0.01) proportion of the tricarboxylic acid (TCA) cycle.

## Discussion

### Microbial Population and Chemical Composition of Material

The desire to reduce feed cost and ensure ruminant production system profitability has prompted the development of WP resources as an alternative source of protein for sustainable animal production. WP are recommended as ruminant feed to improve forage quality, reduce the need to purchase concentrate, and decrease the cost of feeding animals. Generally, animals prefer forage with low fibre content and high NDF digestibility, as well as a high CP content (Egan, 1980), because this type of forage is associated with high animal intake and efficient assimilation of energy, minerals, and vitamins. In this study, as shown in Table 1, the WP material was rich in CP (>23.91% of DM) and protein (74.95% of CP); however, it had low fibre, binding protein, and NDIP contents. In addition, the WP material also had high contents of energy and macro-minerals. Therefore, the WP material contains high levels of digestible feed ingredients for use in livestock feed as a source of protein. It is difficult to produce good-quality silage using WP because of their high moisture content (Du et al., 2021b). In addition, the WSC, LAB, and LBC contents of materials affect silage fermentation (Cai et al., 2020a). The moisture content of WP material was >76%, possibly leading to poor fermentation. When NG and CS were added to WP, the moisture content was <70%, within the range (60–70%) suitable for fermentation (McDonald et al., 1991; Du et al., 2021b). A suitable moisture content inhibits the growth of harmful microorganisms such as *Clostridium* species; it also prevents butyric acid fermentation and protein decomposition (Cai et al., 2020b; Du et al., 2021a). For silage fermentation, other important factors are WSC > 5% of DM, LAB > 5 lg cfu/g of FM, and low LBC (Cai et al., 1999). In this study, the LAB count and WSC content in WP material were below these levels. This indicates that a small number of LAB is associated with poor silage fermentation, thus reducing fermentation quality. In addition, WP material has a high LBC value similar to the value of legume alfalfa, which hampers silage production (Xu et al., 2019). During ensiling, these cations neutralise the organic acids formed by silage fermentation, thus preventing pH reduction. Therefore, preparation of high-quality silage by direct ensiling of WP is problematic. NG and CS are locally available feed resources with good fermentation characteristics, such as low LBC count, high WSC, and optimal DM content. The addition of NG and CS can overcome some of the difficulties in WP silage fermentation.

### Fermentation Characteristics of Silage

Woody plant can usually be harvested multiple times during the rainy season. If it is prepared and stored in the manner used for hay, some nutrients will be lost during drying and storage (Zhang et al., 2019). After drying, the palatability and nutritional value of the leaves for livestock are greatly reduced (Du et al., 2021b). Silage can preserve the nutrients in fresh forage and ensure the supply of animal feed throughout the year (Yan et al., 2019). LAB have important roles in the preparation of silage, converting WSC into lactic acid, lowering the pH value, and inhibiting the growth of harmful bacteria; thus, they produce high-quality silage (Cai et al., 2020a). In this study (Tables 2, 3), particularly in the gliricidia or leucaena + CS (90 + 10) mixed silages, the LAB population significantly increased after 60 days of ensiling, inhibiting the growth of harmful bacteria. Adjustment of the moisture content and enhancement of fermentation substrate levels are methods that improve fermentation quality. Therefore, when using WP to prepare silage, the recommended grass or crop stover proportion is approximately 10%; this should be adjusted to 60–70% depending on moisture content. After harvest, fresh CS is generally rich in LAB and WSC, and the addition of CS can improve the fermentation quality of WP mixture silage. However, NG itself does not have good fermentation characteristics because of its low WSC content and low LAB count. The NG silage and WP + NG mixed silage did not show improved fermentation quality. Therefore, CS is recommended for the preparation of mixture silage with WP in Africa.

### Microbial Community of Material and Silage

Silage fermentation is a dynamic process of microbial community succession and shifts in metabolite levels. Using traditional culture methods, only some microorganisms in silage can be isolated and identified; this hampers the analysis of fermentation mechanisms. Next-generation sequencing (NGS) technology has been used to evaluate silage microbial community, abundance, and diversity. It is a high-throughput approach that can detect a large number of microbial taxa. However, microorganisms can be identified only to the genus level, and the complete microbial community cannot be profiled based on partial 16S rRNA gene sequences (Bokulich and Mills, 2012; Mayo et al., 2014). The SMRT sequencing technology used in this study enables accurate evaluation of the microbial community and diversity within silage (Xu et al., 2019). As shown in Figure 1, compared with the NG and CS materials, the greater OTU numbers in gliricidia and leucaena may reflect epiphytic microorganisms on the surface, because of the high moisture content and rich nutrition of fresh branches and leaves. After ensiling, natural LAB create an acidic environment to inhibit core microorganisms, reducing their abundance and forming a unique microbial community in the silages.

Microbial community structure and function affect silage fermentation (McEniry et al., 2010; Guan et al., 2018; Cai et al., 2020b). As shown in Figure 2, the Gram-negative bacterium *P. agglomerans* was the major species in WP materials. This plant pathogen is often present in forage and is considered harmful in silage (Du et al., 2021a). The dominant species in the NG and CS materials was *M. trichothecenolyticum*, a Gram-positive and aerobic or weakly anaerobic non-spore-producing and acid-fast species. In contrast, the abundance of *L. plantarum* was low in all materials, indicating that WP were similar to forage and crop by-products; WP typically have a low abundance of LAB but a high abundance of undesirable bacteria before ensiling (Tian et al., 2020; Du et al., 2021b). Therefore, measures to control the microbial community are needed to improve silage fermentation.

Silage fermentation is conducted in an anaerobic, closed, solid-fermentation system (Cai et al., 1998). A higher relative abundance of LAB is frequently observed in this system, and the different microbial community structure have differential effects on the silage fermentation (Queiroz et al., 2013; Cai et al., 2020b). In this study (Figure 3), when WP and CS were mixed to prepare WP silage, the LAB can quickly become dominant bacteria and complete the succession process from Gram-negative bacteria to Gram-positive bacteria, indicating that mixture silage can effectively improve the fermentation quality by improving the microbial community. However, there was not great difference between the microbial communities in the NG silage and WP + NG mixed silages. A reasonable explanation for this phenomenon is that, compared with NG, CS contains more lactic acid bacteria, such as *L. plantarum*, which have stronger fermentation ability and acid tolerance. In the process of mixture silage fermentation, these epiphytic LAB existing on CS can quickly respond to the double pressure of anaerobic and acidic silage environment, carry out lactic acid fermentation and improve the fermentation quality of silage (Wang et al., 2019; Du et al., 2021a).

### Interaction Between Bacterial Community and Fermentation Product

Microorganisms affect silage fermentation through a series of metabolites. For example, *Lactobacillus* species use WSC to produce lactic acid, while *Enterobacter* species ferment lactic acid into products such as acetic acid (McDonald et al., 1991), thereby influencing silage fermentation quality and aroma. In addition, these metabolites affect the microbial community structure. For example, lactic acid and acetic acid lower the pH and inhibit the growth of aerobic bacteria and moulds, thus affecting the aerobic stability of silage. Accordingly, silage fermentation is modulated by microbial community structure and metabolites, which interact during fermentation.

In this study, the production of lactic acid was attributed to *Lactobacillus* species. Lactobacilli are homofermentative type LAB that inhibit the activities of harmful microorganisms by rapid acidification in the later stage of silage fermentation (Cai, 2004; Mu et al., 2020). In the Figure 4, *L. plantarum, L. brevis, L. fermentum, and W. paramesenteroides* grew well and produced lactic acid in silage, inhibiting the growth of *P. agglomerans* by reducing the pH. The *A. muciniphila* competed for nutrients with *L. plantarum* during ensiling, while degrading proteins to produce NH_3_-N. However, silage fermentation by *L. plantarum* inhibited the growth of these bacteria and the production of NH_3_-N.

In the Figure 5, during silage fermentation, *L. plantarum* was positively correlated with *M. trichothecenolyticum* and negatively correlated with both *K. cowanii* and *C. granulosa*. The nature of this relationship is unclear; however, *M. trichothecenolyticum* is dependent on respiratory metabolism that produces acid from glucose and other sugars during the ensiling process. Therefore, *L. plantarum* inhibits the growth of other Gram-negative bacteria; it may promote the growth of *M. trichothecenolyticum*. The abundances of *K. cowanii* and *C. granulosa* were negatively correlated with the abundance of *L. plantarum*. These bacteria are often distributed on the surface of forage and compete with LAB for nutrients in the early stage of silage fermentation. As silage fermentation progresses, they are inhibited by the lactic acid from LAB. The *P. agglomerans* and *P. pasteri* are frequently present in silage fermentation; they produce n-butyric acid, acetic acid, propionic acid, isobutyric acid, isovaleric acid, and phenylacetic acid, thus reducing fermentation quality. Therefore, the two bacteria are positively correlated with each other, but both are negatively correlated with *L. plantarum*.

### Kyoto Encyclopedia of Genes and Genomes Metabolism Pathway Analysis

In the KEGG analysis, carbohydrate metabolism was the most important metabolic category in gliricidia materials and silage. Pyruvate metabolism, glycolysis, and butyrate metabolism were predominant components of the carbohydrate metabolic pathways, consistent with the main silage metabolism pathway reported by Du et al. (2021b). In this study (Figure 6), silage had a higher proportion of carbohydrate metabolism pathways than did the corresponding material, reflecting the community dynamics and metabolic activities of the major LAB. Amino acid metabolism can be oxidised into carbon dioxide and water through the tricarboxylic acid cycle, releasing energy (Halperin and Jungas, 1983). Therefore, fresh materials have higher rates of amino acid metabolism and energy metabolism pathways than do silages.

The roles of secondary metabolites and antibiotics in silage are unclear. Secondary metabolites and antibiotics may be produced by microorganisms—including bacteria, fungi, and actinomycetes—during silage fermentation, which interfere with the development of other cells. LAB grew more vigorously in mixture silage than in gliricidia silage, inhibiting the growth and metabolic activities of other microorganisms while suppressing the biosynthesis of secondary metabolites and antibiotics. However, the proportion of citric acid cycle was higher in the gliricidia + CS mixed silage than in gliricidia silage. In the citric acid cycle, the condensation reaction between oxaloacetate and the acetyl group of acetyl-CoA produces citric acid. Citric acid is used as a food acidifier, flavouring agent, and chelating agent. Citric acid-mediated adjustment of pH value can improve the performance of antioxidants, inhibit the activity of enzymes, and extend the shelf life of food (Ke et al., 2017). The WP and CS mixture silage has an increased citric acid content, which will improve its flavour and quality, facilitating long-term storage.

## Conclusion

To improve fermentation quality and develop a preparation method for nutrient-rich WP, we used SMRT sequencing technology to study the microbial community and fermentation characteristics of WP silage following the addition of NG and CS in Southern Africa. WP have high CP, energy, and mineral contents; they offer ideal feed for ruminants. However, the preparation of good-quality WP silage is hampered by the low WSC content, low LAB count, and high LBC in WP. In PM silage prepared with NG and CS, the dominant microbial community shifted from Gram-negative bacteria to LAB, promoting lactic acid fermentation. *L. plantarum* was predominant, which improved silage flavour and quality. CS has good silage characteristics, helping to overcome the influence of unfavourable factors in silage fermentation. Addition of CS improves the microbial community structure and metabolic pathways of silage; thus, the combination of CS and WP is suitable for high-quality silage. WP offer a potential source of high-protein feed for ruminants, which can alleviate the feed shortage in Southern Africa.

## Data Availability Statement

The original contributions presented in the study are included in the article/supplementary material, further inquiries can be directed to the corresponding author.

## Author Contributions

ZD and YC conceived and designed the study. ZD, SY, and TO carried out the experiments. ZD and DN carried out the data analysis. ZD, YC, DE, BT, and FM wrote and revised the manuscript. All authors read and approved the manuscript.

## Conflict of Interest

The authors declare that the research was conducted in the absence of any commercial or financial relationships that could be construed as a potential conflict of interest.

## Publisher’s Note

All claims expressed in this article are solely those of the authors and do not necessarily represent those of their affiliated organizations, or those of the publisher, the editors and the reviewers. Any product that may be evaluated in this article, or claim that may be made by its manufacturer, is not guaranteed or endorsed by the publisher.

## Abbreviations

WP: woody plant
PM: paper mulberry
LBC: lactic acid buffer capacity
WSC: water-soluble carbohydrate
DM: dry matter
FM: fresh matter
OM: organic matter
CP: crude protein
EE: ether extract
NDF: neutral detergent fibre
ADF: acid detergent fibre
ADL: acid detergent lignin
NH_3_-N: ammonia nitrogen
LAB: lactic acid bacteria
OTU: operational taxonomic unit
cfu: colony-forming unit
NG: Napier grass
CS: corn stover.
